# Next‐Generation Cancer‐Specific Hybrid Theranostic Nanomaterials: MAGE‐A3 NIR Persistent Luminescence Nanoparticles Conjugated to Afatinib for In Situ Suppression of Lung Adenocarcinoma Growth and Metastasis

**DOI:** 10.1002/advs.201903741

**Published:** 2020-03-14

**Authors:** Ming‐Hsien Chan, Wen‐Tse Huang, Jing Wang, Ru‐Shi Liu, Michael Hsiao

**Affiliations:** ^1^ Genomics Research Center Academia Sinica Taipei 115 Taiwan; ^2^ Department of Chemistry National Taiwan University Taipei 106 Taiwan; ^3^ Ministry of Education Key Laboratory of Bioinorganic and Synthetic Chemistry State Key Laboratory of Optoelectronic Materials and Technologies School of Chemistry Sun Yat‐Sen University Guangzhou Guangdong 510275 China; ^4^ Department of Mechanical Engineering Graduate Institute of Manufacturing Technology National Taipei University of Technology Taipei 106 Taiwan; ^5^ Department of Biochemistry College of Medicine Kaohsiung Medical University Kaohsiung 807 Taiwan

**Keywords:** afatinib, aptamer, cancer therapy, lung cancer metastasis tracking, persistent luminescence nanoparticles

## Abstract

The rate of lung cancer has gradually increased in recent years, with an average annual increase of 15%. Afatinib (AFT) plays a key role in preventing non‐small cell lung carcinoma (NSCLC) growth and spread. To increase the efficiency of drug loading and NSCLC cell tracking, near infrared‐persistent luminescence nanomaterials (NIR PLNs), a silica shell‐assisted synthetic route for mono‐dispersal, are developed and used in the nanovehicle. After optimizing their physical and chemical properties, the NIR PLNs are able to absorb light energy and emit NIR luminescence for several hours. In this research, NIR PLNs are functionalized for drug‐carrying capabilities. Effective accumulation of target drugs, such as AFT, using PLN nanomaterials can lead to unique anticancer therapeutic benefits (AFT‐PLN). To minimize side effects and increase drug accumulation, nanomaterials with targeting abilities are used instead of simple drugs to inhibit the growth of tumor cells. Thus, the specific targeting aptamer, MAGE‐A3 (MAp) is identified, and the PLN to increase its targeting ability (AFT‐PLN@MAp) accordingly modified. The advancement of nanoscale techniques in the field of lung cancer is urgently needed; this research presents a plausible diagnostic strategy and a novel method for therapeutic administration.

## Graphical abstract

The five‐year survival mortality rate of lung cancer remains only 5–8%. By suppressing the signaling of the epidermal growth factor receptor, the afatinib‐based drug delivery system plays a key role in preventing tumor growth and spread. Modifying with targeting aptamer, persistent luminescence nanomaterials can load afatinib as a specific drug vehicle to track and inhibit lung adenocarcinoma growth.

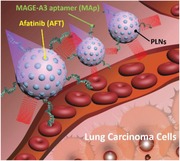

## 1 Introduction

Lung cancer is the major cause of cancer‐related deaths in the world; the treatment options for non‐small cell lung cancer (NSCLC) are based mainly on the stage of cancer.^[^ ^1^ ^]^ Once lung cancer progresses to stage III, the survival rate is only 15%. This is because the tumor cells have metastasized to the mediastinal lymph nodes, or are about to expand to the adjacent esophagus, pleura, chest wall, pericardium, or aponeurosis. Lung cancer is responsible for more deaths than breast, colon, and prostate tumors combined. A current problem in the treatment of lung cancer is the inability to do specific targeting or to trace targeting drugs for effective therapy. There are few studies on a preclinical in situ lung adenocarcinoma (LUAD) model treatment. Thus, therapeutic intervention for an effective and safe LUAD diagnosis and therapy are still challenging. Developing multi‐functional nanoplatforms to sense cancer and drug delivery systems are important for tumor biology applications. For example, fluorescent nanomaterials have high resolution and can track specific cancer cells in blood circulation to facilitate diagnosis.^[^ ^2^, ^3^, ^4^ ^]^ Near‐infrared persistent luminescence nanoparticles (NIR PLNs) have a special nonlinear radiation process, involving the absorption and retention of photons for several hours, followed by the emission of long‐term luminescence that lasts for at least 1 to 2 h.^[^ ^5^, ^6^, ^7^ ^]^ For biological application, NIR persistent luminescence materials have recently attracted increasing academic attention because of their potential as novel optical contrast agents for in vivo bioimaging within the NIR window. NIR PLNs have a high signal‐to‐noise ratio for luminescence detection or imaging within an organism because most proteins or organelles emit a luminescence signal with an ultraviolet or visible emission; therefore, these nanomaterials are free from background auto‐fluorescence.^[^ ^8^ ^]^ Additionally, the persistent luminescence of NIR within the tissue transparency window (650–1350 nm) can be detected for hours after the termination of excitation.^[^ ^9^ ^]^ The core of NIR PLNs comprises oxides of transition metals and some other metal elements, such as zinc (Zn) and gallium (Ga), which can generate the host materials of ZnGa_2_O_4_ (ZGO). The key to generating long‐term luminescence is chromium (Cr^3+^)‐doped persistent material. Cr^3+^ ions can be obtained by tailoring the trap distribution and crystal field in the host material.^[^ ^10^ ^]^ Because of the long‐term illuminating effect and luminescence ability, PLNs can effectively track the movement of cancer cells. For PLNs, the major problem in biological application is the relationship between “scale” and “luminescence performance.”^[^ ^11^ ^]^ Small PLNs (<20 nm) have a poor persistent luminescence performance, in contrast to large PLNs, which have excellent NIR persistent luminescence (bulk, usually >1 µm).^[^ ^12^ ^]^ In the current study, methods to control PLNs with ceria are described. The sintering of two different crystal phase structures can confine the PLN size to obtain uniformity. The mesoporous silica nanoparticle combines various metal elements to limit the growth environment of the PLNs. Additionally, because of the confinement effect provided by the mesoporous structure, crystallinity can be improved; thus, the mesoporous structure provides great potential for the loading of specific anticancer drugs. In eastern Asian society, lung cancer has always been regarded as the main target “tumor combat.”^[^ ^13^ ^]^

Five‐year survival from lung cancer drastically decreases when lung cancer patients are diagnosed with metastatic disease, indicating that lung cancer metastasis is the primary contributor to cancer‐related death.^[^ ^14^ ^]^ In the current treatment of lung cancer, the use of first‐line targeting drugs targets the weak points (usual oncogenes) of cancer cells in different patients and provides them with the best therapeutic effect and minimal side effects.^[^ ^15^ ^]^ Many cancer cells continue to emit signals that stimulate cell growth due to mutations in the receptor proteins on the cell surface (such as epidermal growth factor receptor (EGFR)) or various expression patterns. Target drugs are expected to bind these abnormal receptor proteins, inhibit their activity, and inhibit cancer cell growth.^[^ ^16^, ^17^, ^18^ ^]^ Therefore, anticancer drugs such as afatinib (AFT), which irreversibly binds to the ErbB family of receptors (including four different cancer cell receptors EGFR, HER2, ErbB3, and ErbB4), have become targets for mutant EGFR challenge.^[^ ^19^ ^]^ A highly effective drug blocks the growth of cancer cells. However, different classes of drugs can cause diverse kinds of side effects. The most common side effects are diarrhea and decreases in liver function; others, such as body weakness, elevated blood pressure, lighter hair color, skin, and nail variation, may also occur because the drug itself also partially acts on normal cells, causing side effects in the patient.^[^ ^20^ ^]^ In this study, we employed a nanocomposite with a surface modification as an AFT delivery system where a cell‐specific targeting moiety was conjugated onto the PLN nanocarrier. The challenges of developing theranostics for lung cancer metastasis treatment include determining a specific receptor and ensuring the biological safety of the multi‐functional nanocarrier.^[^ ^21^, ^22^, ^23^ ^]^

The novel PLN, which was conjugated to a specific aptamer sequence named the MAGE‐A3 aptamer (MAp), produces a non‐toxic nanoplatform that can effectively accumulate in lung cancer cells.^[^ ^24^ ^]^ This DNA aptamer was identified against the tumor‐specific melanoma‐associated peptide antigen (MAGE‐A3111‐125).^[^ ^25^, ^26^ ^]^ Based on the above description, the development of novel targeting methods is indispensable for tumor tracking. As a “bio‐missile,” the multifunctional nanocarrier could release AFT to inhibit the growth of tumor cells. The MAp recognizes the MAGE‐A3111‐125 receptor to work synergistically to achieve the greatest therapeutic effect.^[^ ^27^ ^]^ First, a long afterglow material based on mesoporous silica was synthesized. The successfully synthesized PLN material was irradiated with UV light in vitro and emitted red and infrared light for several hours. With this unique property, long‐lasting materials could track the changes and metabolism of the tumor for a long period of time. Next, AFT, used in first‐line target therapy, was loaded into the mesopores (AFT‐PLN). The 3 to 5 nm mesoporous material is very suitable as a delivery vehicle due to its small size; it can continuously release drugs over 12 h.^[^ ^28^, ^29^, ^30^ ^]^ Finally, we expect that the specific aptamer molecule, MAp, could be modified on the surface of the material so that the entire system will be actively transported to the tumor by specific binding (AFT‐PLN@MAp). This “MAp” aptamer is essentially a piece of the map that leads the nanomaterials to find the location of the tumor cells.^[^ ^31^ ^]^ Additionally, we evaluated mouse models with different modes of administration. It is expected that sample injections could be performed using intratracheal (IT), subcutaneous (SC), and intravenous (IV) injections to evaluate the response of mice to different conditions of drug administration. According to the experimental results, AFT‐PLN@MAp was successful in suppressing in situ LUAD growth and metastasis, suggesting its promise to be developed into a clinical therapy regimen to treat advanced lung cancer patients.

## 2 Results and Discussion

### 2.1 Material Identification

**Figure 1:**
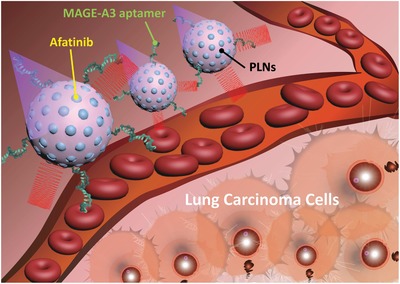
A scheme of AFT‐PLN@MAp as a targeting anticancer nanoplatform. A potential strategy of AFT‐PLN@MAp is provided for diagnostic and therapeutic treatment of metastasis in lung cancer. Current therapeutic methods intending to cure the metastasis of tumor cells have major issues. This nanoplatform can be used with a MAp to target specific aptamers; PLNs are actively transported to tumor cells. PLNs embedded with AFT target drugs can effectively treat tumor cells, limiting cancer cell metastasis.

**Figure 2:**
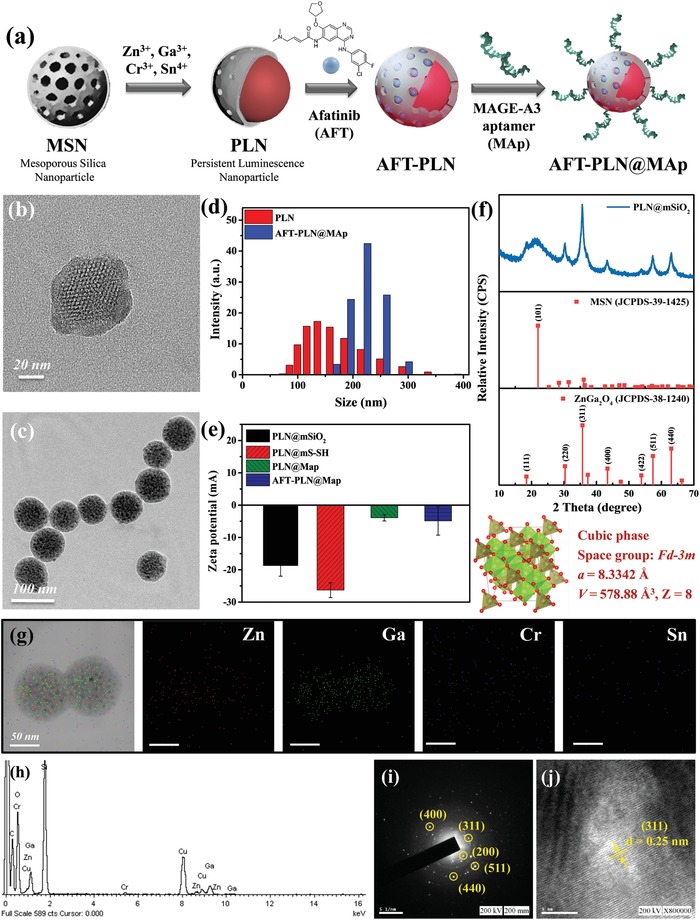
Basic characteristics of PLNs and the derivative PLNs. a) Schematic diagram of the synthetic AFT‐PLN@MAp procedure. This study first used mesoporous silica nanoparticles (MSN) as a material template and added inorganic metal molecules to the core of the hole to form a PLN. After washing the metal ions, the AFT drug molecules are loaded and the aptamer is modified to form AFT‐PLN@MAp. Transmission electron microscopy images of b) MSN and c) PLNs, which form a deep black dot‐like ZGOCS structure in the MSN of the original hole structure. d) Dynamic light scattering and e) zeta potential data showing the degree of dispersion and surface electrical properties of the material, respectively. The PLN has a hydration radius of approximately 125 nm and an AFT‐PLN@MAp size of approximately 225 nm. Additionally, AFT‐PLN@MAp has a suitable degree and physiological charge of dispersion and a more concentrated particle size distribution. f) X‐ray powder diffraction (XRD) data. JCPDS‐38‐1240 has a crystal phase structure consistent with the synthesized AFT‐PLN@MAp. Energy‐dispersive X‐ray spectroscopy g) mapping and h) spectrum. The elemental structure of the material was tested using Zn, Ga, Cr, and Sn. Selected area (electron) diffraction data with i) specific area and j) d‐spacing were compared with the data collected by XRD, where the (311) characteristic peak is consistent with the XRD.

The primary goal of this study was to develop a nanoscale‐diagnostic treatment platform that can track cancer changes over time and effectively inhibit tumor metastasis. Even better, if the tumor metastasizes, the PLN material can track the metastasis of cancer cells or inhibit the metastatic process of tumor cells. The multifunctional PLN nanocarrier was fabricated to be used in theranostic applications.^[^ ^32^ ^]^ This NIR PLN, attached to the specific aptamer ligand MAp, serves as an efficient drug carrier. We tested the nanocomposite in an in vitro lung cancer cell model and in vivo mouse model (**Figure** **1**). As a material that can emit fluorescent signals for a long period of time, NIR PLNs are inorganic materials that can have pore structure and are synthesized via a high‐temperature sintering method. The preparation steps for the entire material are illustrated in **Figure** **2a**. First, MSN nanospheres were prepared to limit the growth size of the PLN materials. The suitable pore radius accommodated AFT drugs in the tunnel. Next, the constituent metal ions of the PLN were added, and the entire material was synthesized by high‐temperature sintering to synthesize PLN. After calcination was completed, the PLN material was washed and removed as a precursor to the reaction. This material has an unfilled mesoporous structure that can be used for AFT drug loading (AFT‐PLN). Finally, an SH‐modified aptamer, MAp, was used to connect AFT‐PLN and form AFT‐PLN@MAp by a disulfide bond. The original MSN was approximately 50 nm in size and had a distinct pore structure (Figure 2b). After sintering at high temperature, the holes grew into a dark‐colored ZnGa_2_O_4_:Cr^3+^,Sn^4+^ (ZGOCS) core. Calcination was performed at 1000 °C to make PLNs. From the TEM image shown in Figure 2c, ZGOCS nanoparticles (dark spots) can easily distribute in the nanoscale pores of the MSN (ZGOCS@MSN, also named PLNs).

In addition to confirming the size of the material by TEM, we tested its dynamic light scattering (DLS) to confirm the true dispersion of the material in aqueous solution. Figure 2d shows PLN without a drug (AFT) and targeting ligand (MAp) at approximately 150 nm, with partial aggregation. After loading AFT and the modified MAp, the size of AFT‐PLN@MAp increased to 225 nm and its monodispersion was better than that of PLNs. At the same time, the Zeta potential (Figure 2e) was tested and the charge change was monitored in the nanoparticles. The original PLN material (PLN@mSiO_2_) was negatively charged (−19 eV) due to the presence of Si‐OH groups on its surface. To bind the aptamer via disulfide bonding, PLN@mSiO_2_ of the SH group was modified, and the charge of —SH was −26 eV. After conjugation with the aptamer, the surface electrical property rose to around ‐4 eV and was almost electrically neutral when loading AFT (AFT‐PLN@MAp, −5 eV), indicating the surface electrical properties during the entire synthesis process. To confirm the crystal structure of PLNs, X‐ray diffraction (XRD) was used to determine whether the crystal structure of the PLN material changed (Figure 2f). Compared with ZGO's JCPDS card 38–2140, PLN@mSiO_2_ was produced. Outside the SiO_2_ structure of the amorphous phase (approximately 20°), the remaining peaks were consistent with the standard. The cubic phase was presented.^[^ ^33^ ^]^ Additionally, the energy dispersive spectrometer (EDS, Figure 2g) data confirmed that the black particles in the holes were PLNs and that the Zn and Ga signals were consistent with the TEM images. The Zn to Ga ratio shown in Figure 2h is approximately equal to 1:2, confirming that the ZGO material was formed in the MSN. Furthermore, by analyzing the crystal structure of PLN by selected area electron diffraction (SAED), a diffraction pattern conforming to XRD was obtained. The unique structure of PLN with d spacing = 0.25 nm on the (311) surface confirmed the integrity of the material synthesis (Figure 2i,j). The morphology of MSN and AFT‐PLN@Map was coated with a Pt layer and examined by scanning electron microscope (SEM) in the detailed work under 5 kV electron beam (Figure S1a,b, Supporting Information). Those maps modified with PLN by disulfide bonds were also confirmed by FTIR data showing 814 and 2364 cm^−1^ —SH functional group (Figure S1c, Supporting Information).

### 2.2 Optical Analysis of PLNs

**Figure 3:**
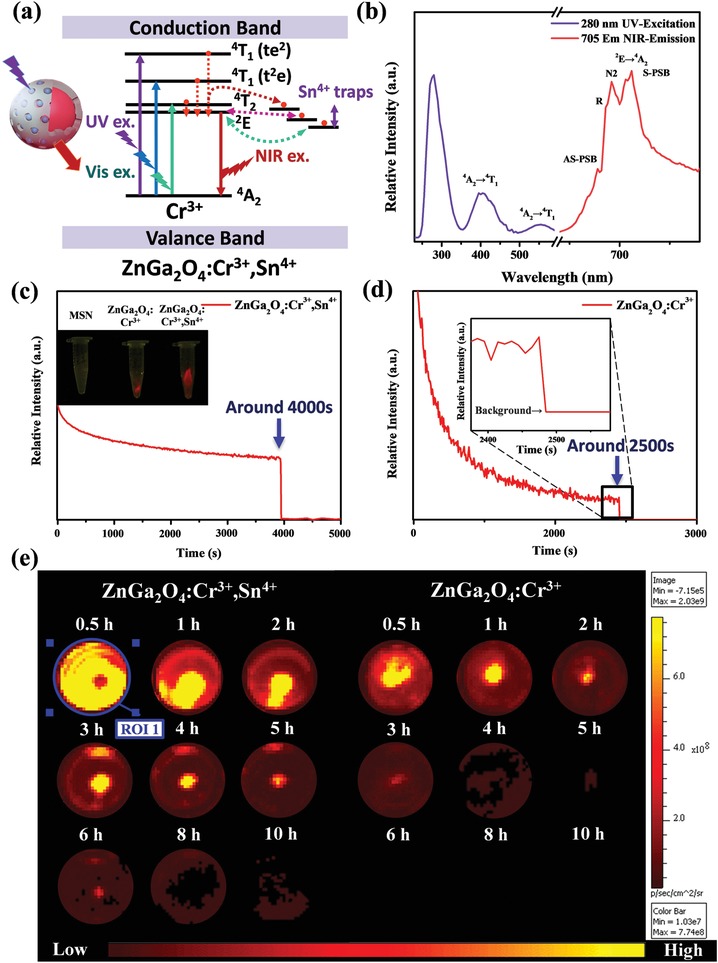
Different compositions of PLNs can affect optical physiognomies. a) Scheme energy diagram using PLNs. Cr^3+^ and Sn^4+^ have the ability to delay electron emission. b) Stimulation of PLNs from under 250 to 565 nm leads to the emission of particles during UV irradiation and generates red to NIR luminescence. The lifetime results of c) ZGOCS (inset: afterglow images taken 1 min after UV irradiation) and d) ZGOC (inset: the amplified spectrum shows that the pure doping of Cr^3+^ has a long afterglow capability). The persistent luminescence signal can be detected by IVIS. The images were acquired 5 min and 10 min after UV exposure. The slice remained on top of the e) ZGOCS and ZGOC throughout the experiment. The test time was from 0.5 h to 10 h. The blue circle is a region of interest (ROI); the same regions were applied for all testing points.

The difference in the energy levels of transition elements and lanthanides allows PLNs to shine at different wavelengths.^[^ ^34^ ^]^ Depending on the transition and/or lanthanide doping, PLNs can emit luminescence from visible to far‐infrared light.^[^ ^35^ ^]^ These elements are doped at different proportions so that the material can have a broad emission (**Figure** **3a**). Here, the mesoporous silica nanoparticles (MSNs) were loaded with ZnGa_2_O_4_:Cr^3+^ to generate PLN. NIR luminescence imaging was proposed and successfully prepared. The excitation band of PLN is located at 265 nm; the transfer band of O_2_‐Ga^3+^ in the ZnGa_2_O_4_ host could attribute 310, 420, and 560 nm to the ^4^A_2_→^4^T_1_(^4^P), ^4^A_2_→^4^T_1_(^4^F), and ^4^A_2_→^4^T_2_(^4^F) transitions of Cr^3+^, respectively (Figure 3b). Because of the overlap with the transfer band of the ZnGa_2_O_4_ host (265 nm), clearly identifying the ^4^A_2_→^4^T_1_(^4^P) band of Cr^3+^ (310 nm) is difficult. PLN nanospheres were monitored at 695 nm after 265 nm UV light illumination (Figure 3c).^[^ ^36^ ^]^ According to current research, doping Sn^4+^ is beneficial for improving the luminous efficiency and lifetime of PLNs because the adjacent energy levels of Sn^4+^ and Cr^3+^ can form electron traps, which store high‐energy electrons in the excited state for a longer period of time, and the excess energy storage space also improves the luminous efficiency of PLN. As shown in Figure 3c,d, the NIR persistent luminescence decay curve of ZGOC nanoparticles is 0.7 h; after doping Sn^4+^, luminescence will last for up to 1 h. These PLN can be applied as contrast agents for fluorescent imaging with a high spatial resolution and long‐term in vivo imaging with a rechargeable ability. Additionally, they provide sensitive imaging due to their NIR persistent luminescence properties. PLNs can be tuned to emit a specific luminescence by doping with different elements. They also exhibit excellent NIR persistent luminescence and hold the potential for targeting due to the silica surface having many free sites. Moreover, ZGOC nanoparticles were tested using the in vivo imaging system (IVIS) system to evaluate the luminescence intensity and lifetime of ZGOC and ZGOCS nanoparticles. In Figure 3e, it can be clearly seen that ZGOCS nanoparticles have a higher photon content than ZGOC nanoparticles and can be detected for up to approximately 10 h under the IVIS CCD system. ZGOCS nanoparticles had an approximately three times higher luminescence intensity and two times higher maintenance time compared to ZGOC nanoparticles. Besides qualitative progress, quantity optimization was also important. We tuned the Cr^3+^ and Sn^4+^ concentration for doping in ZnGa_2_O_4_. The best doping ratios of Cr^3+^ (Figure S2a and S2b) and Sn^4+^ (Figure S2c and S2d) with the highest emission intensity were evaluated. We collected the data and arranged all region of interest (ROI) numbers into a bar chart to quantify the IVIS data based on Figure 3e (Figure S2e, Supporting Information).

### 2.3 AFT Drug Loading and MAp Targeting Analysis

**Figure 4:**
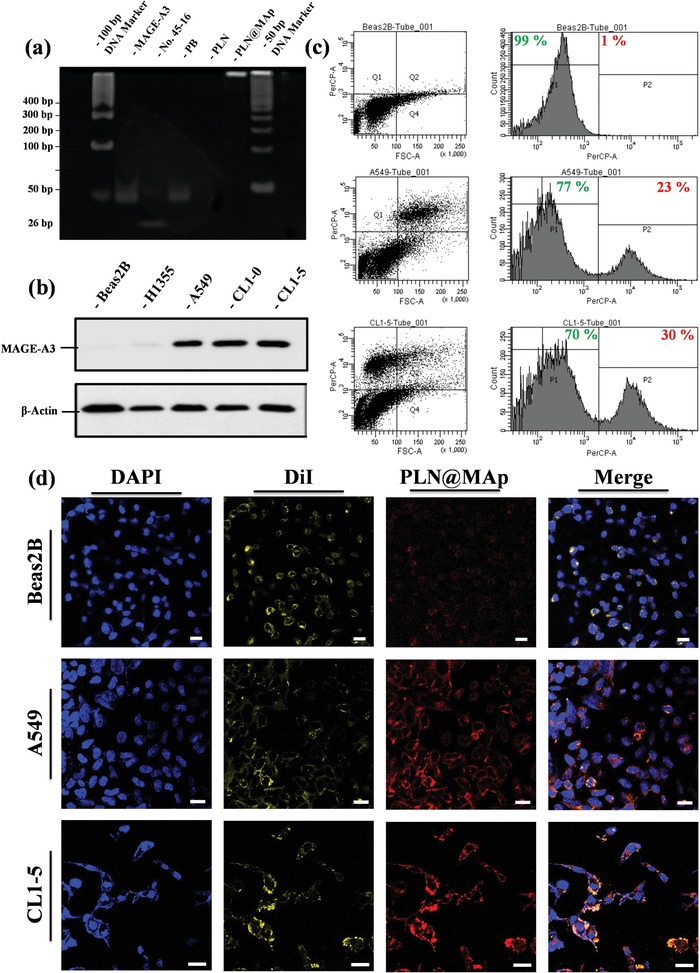
Evaluation of MAp targeting ability. a) DNA gel of MAp analysis. The experimental group from left to right: 100 bp DNA marker, MAp, No. 45‐16 (active site, negative control), PB (52‐mer, positive control), PLN@SiO_2_ (negative control), and PLN@MAp with 50 bp DNA marker. b) Expression of the MAGE‐A3 protein. Western blot results showing the amount of MAGE‐A3 protein signal in Beas2B, H1355, A549, CL1‐0, and in CL1‐5 cells. c) Flow cytometry of PLN@MAp‐treated Beas2B, A549, and CL1‐5 cell lines. The left column is the cell point distribution map, and the fluorescence of the cells can be observed in the A549 and CL1‐5 cells. There is an upward trend of luminescence in the cell lines. The filter selected for testing is PerCP‐A. d) LSCM imaging of PLN@MAp. The blue fluorescence (440 nm) is DAPI staining of the nucleus, yellow fluorescence (570 nm) is DiI staining of the cell membrane, and red luminescence (750 nm) is PLN@MAp itself releasing long‐lasting infrared light. Beas2B, A549, and CL1‐5 cell lines were tested separately (scale bar: 50 µm).

After examining the optical properties of PLN, we first analyzed the mesoporous structure of MSN and PLN. In this study, MSN was used to limit the crystallization environment to form PLN. The BET analysis was performed on the mesoporous structure of MSN, PLN, and AFT‐PLN@MAp, respectively. (Figure S3a,b,c, Supporting Information). Compared with AFT‐PLN@MAp, as shown in Figure S3d, Supporting Information, MSN has a well‐defined structure and belongs to the Type IV structure of BET analysis. The pore size is measured by BET to be approximately 2 nm. However, after PLN crystal synthesis in the pores of MSN, it can be seen that the volume of pores becomes smaller. Furthermore, AFT‐PLN@MAp does not have a porous structure, confirming that the pore structure of MSN has been filled by ZGOCS nanoparticles and AFT (Figure S3e, Supporting Information). To test the weight of AFT drug dose and the modified MAp, the TGA analysis was used to weigh AFT‐PLN@MAp results. In Figure S3f, Supporting Information, it is observed that MSN and pure PLN had almost no tendency to decrease in weight. The groups of AFT‐PLN and PLN‐Map also revealed a weight decease at different temperatures, while AFT‐PLN@MAp showed a tendency to decrease in weight at two stages, 250 and 350 °C. This TGA curve is consistent with the pure AFT TGA test (Figure S1d, Supporting Information), which confirmed AFT has been loaded into the AFT‐PLN@MAp structure. According to the TGA analysis of drug molecules and DNA molecules, the weight drop from 150 to 250 °C, can speculate the load amount of AFT is approximately 15% and that between 250 and 350 °C may be the modified MAp (approximately 10%). To confirm whether the targeting ability of the aptamer is sufficient for use as a next‐generation marker molecule, the MAp used as the target aptamer with the sequence 5′‐ATC CAG AGT GAC GCA GCA AGC ACT CAA TAT TCC CTG GAC ACG GTG GCT TAG T‐3′ was selected.^[^ ^31^ ^]^ In **Figure** **4a**, the MAp sequence is verified against the DNA of the remaining specific segments. The numbers No. 45‐16 are the main site of action of MAp, and PB is the 52‐mer sequence 5′‐ATA GGA GTC GAC CGA CAC AGC ACT CAA TAT TCC CGT CTA CAT CTA AGC TCA T‐3′. The MAp and PB signals appeared at approximately 50 bp in DNA colloidal electrophoresis, and the No 45‐16 signal appeared at 26 bp. Additionally, PLN@MAp was tested for DNA‐binding signals. However, because the material was too big to separate from the MAp position, a bright band only appeared at the beginning of the gel. MAGE‐A3 is highly expressed in tumor tissues; it is 74% more expressed in esophageal cancer tissues and approximately 40% more expressed in lung cancer tissues than in normal tissues. Additionally, intestinal cancer, breast cancer, and head and neck cancer have a high degree of MAGE‐A3 expression.^[^ ^31^ ^]^ Currently, the strategy of the aptamer designed to MAGE‐A3 is still not optimized, as this design is a very advanced method. As shown in Figure 4b, the normal cell line Beas2B did not contain significant MAGE‐A3 protein, and other normal cell lines, such as H1335, did not contain a greater amount of MAGE‐A3 protein. Conversely, the adenocarcinomic cell line, A549, showed significant MAGE‐A3 protein expression, and the malignant and metastatic cell lines CL1‐0 and CL1‐5 showed high MAGE‐A3 protein expression. Different concentrations of MAp on the surface of the PLN have different targeting capabilities for A549 cells. MAp, with an 260 nm absorbance of 10 OD, was added to PLN using 67.5 µL, 125 µL, 250 µL, 500 µL, and 1 mL (Figure S4a–e, Supporting Information), respectively. A 500 µL and 1 mL MAp approximated a similar targeting capability from the percentage of the flow plot. As the concentration of aptamer added to PLN became lower, the concentration of PLN accumulating in A549 cells decreased, indicating that the targeting effect of MAp may have occupied most of the space on the PLN at 500 µL. This was similar to the observed targeting when 1 mL MAp was added (Figure S4f, Supporting Information). PLN@MAp was formed by modifying the MAp on the PLN and was tested by flow cytometry; PLN@MAp with red to near‐infrared light could be used as a biological probe to label cancer cells. In Figure 4c, the normal cell line, Beas2B, showed no significant phagocytosis compared with A549 and CL1‐5 cells. The red fluorescent shift of the A549 cell line was nearly 23%, while the CL1‐5 cell line shifted by 30%. Thus, cancer cells have a more pronounced phagocytic effect of PLN@MAp. This data was replicated with laser scanning confocal microscopy (Figure 4d). DAPI was used to stain the nucleus, and DiI was used to stain the cell membrane. After confirming the cell position, PLN@MAp was found in the cytoplasm and near the cell membrane. Additionally, the normal cell line Beas2B, which had low phagocytosis of PLN@MAp, confirmed that MAp could effectively label the location of cancer cells.

### 2.4 Drug Therapy Evaluation Experiment

**Figure 5:**
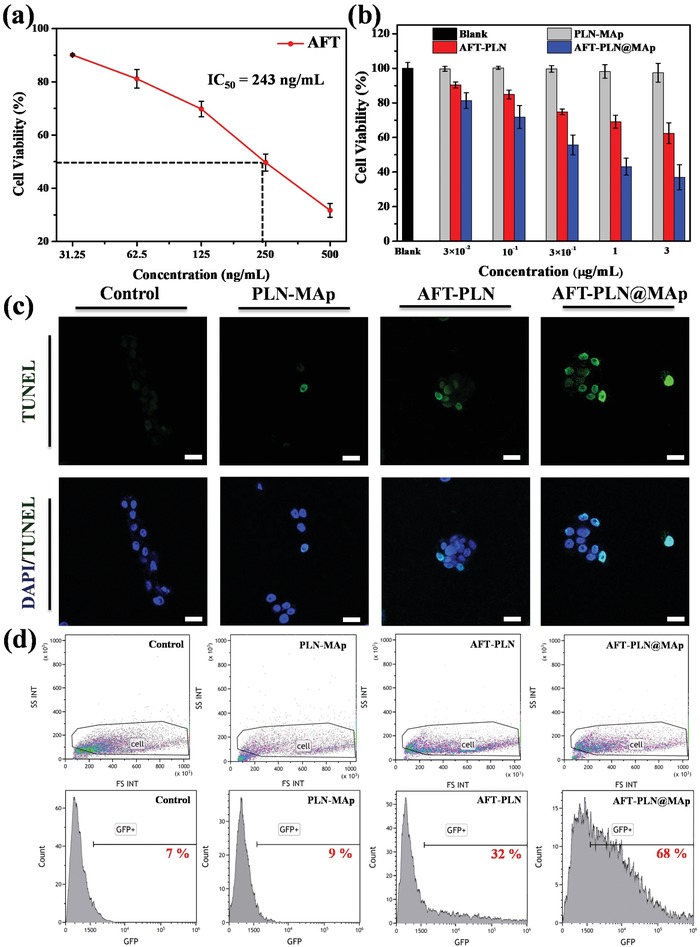
In vitro cell viability and toxicity analysis. a) AFT IC_50_ testing using A549 cells. The concentration of AFT required to reach the IC_50_ dose was approximately 243 ng mL^−1^. b) Cytotoxicity of PLN‐MAp, AFT‐PLN, and AFT‐PLN@MAp. Cells were co‐cultured with A549 cells for 24 h. c) TUNEL assay to detect DNA breaks formed when DNA fragmentation occurs in the last phase of apoptosis. Three groups, PLN‐MAp, AFT‐PLN, and AFT‐PLN@MAp, are analyzed. Blue fluorescence (440 nm) is DAPI staining of the nucleus, and green fluorescence (520 nm) is TUNEL fluorescence (scale bar: 50 µm). d) Flow cytometric analysis of the TUNEL assay. The profile of control cells is gated to fit all different experimental groups: control, PLN‐MAp, AFT‐PLN, and AFT‐PLN@MAp. Fluorescence‐activated cell sorting profiles of cell distribution and green fluorescence protein fluorescence indicate the TUNEL^+^ percentage of cells in the assay.

AFT is an innovative anticancer treatment and a new generation of oral drugs that specifically target cancer cells. AFT is the first drug shown to irreversibly bind to the ErbB family of receptors (including four different cancer cell epidermal growth factors EGFR, HER2, ErbB3, and ErbB4). AFT is an anticancer treatment that more effectively and specifically blocks the signals that trigger cancer cell growth, reducing or delaying the proliferation of cancer cells. AFT has been approved as a first‐line daily oral treatment in many countries, including the United States, Europe, and Taiwan, for NSCLC with EGFR mutant cancer cells. By suppressing the signaling of the above ErbB family members, AFT plays a key role in preventing tumor growth and spread. Because AFT irreversibly binds to the ErbB family of receptors, it disrupts downstream signaling, prevents cancer cell growth, and induces apoptosis (programmed death) in cancer cells. Thus, the irreversible binding properties of AFT to the ErbB family of receptors provides a more durable, selective, covalent, and complete disruption of the transmission of information in cancer cells, resulting in unique characteristics compared with other drugs that target specific cells. This anticancer treatment has the potential to inhibit a wide range of tumor cell growth, and its efficacy is significant. The absorption spectrum and release curve of AFT is shown in Figure S5a,b, Supporting Information. In the drug release experiment, we used secondary water with pH = 7 to test whether the surface‐modified polymer, such as a dendrimer or an aptamer, can slow the rapid release of the drug. The concentration of 100 ng mL^−1^ of AFT and 670 ng mL^−1^ of AFT‐PLN was compared with that of AFT‐PLN@MAp. AFT loading is approximately 15%, so the same concentration is used for comparison. AFT‐PLN@MAp has an aptamer steric barrier that allows the drug to be released into the solution slowly, making AFT‐PLN@MAp more competitive and prolonging the release time of the drug. However, because no trigger switch was added to completely avoid drug release, most drugs were released into solution after 12 h. For the in vitro study, A549 cells were selected as the cell membrane group. The IC_50_ of AFT for A549 cells is approximately 250 ng mL^−1^, as shown in **Figure** **5a**. The different nanomaterial control and experimental groups, were also tested for their A549 and Beas2B cell compatibility and were revealed to be highly biocompatible (Figure S5c,d, Supporting Information). However, because of the enhanced permeability and retention effect and the modified label aptamer MAp, AFT‐PLN@MAp had a lower IC_50_ (the materials had an IC_50_ of approximately 0.3 µg mL^−1^, at which point the dose was approximately 45 ng mL^−1^ as shown in Figure 5b and Figure S5f, Supporting Information), which demonstrated that carrying drugs with nanomaterials could effectively reduce the amount of drugs used and improve efficacy. At the same time, we used the terminal deoxynucleotidyl transferase dUTP nick end labeling (TUNEL) method to detect apoptosis using fluorescein (FITC). As shown in Figure 5c and Figure S5e, Supporting Information, the most obvious TUNEL fluorescence is demonstrated in the AFT‐PLN@MAp group. These data indicated that active accumulation of AFT in A549 cancer cells by targeted methods could effectively improve treatment of cancer. Figure 5d demonstrates the TUNEL confocal analysis quantified by flow cytometry to digitize the results of the confocal images.

**Figure 6:**
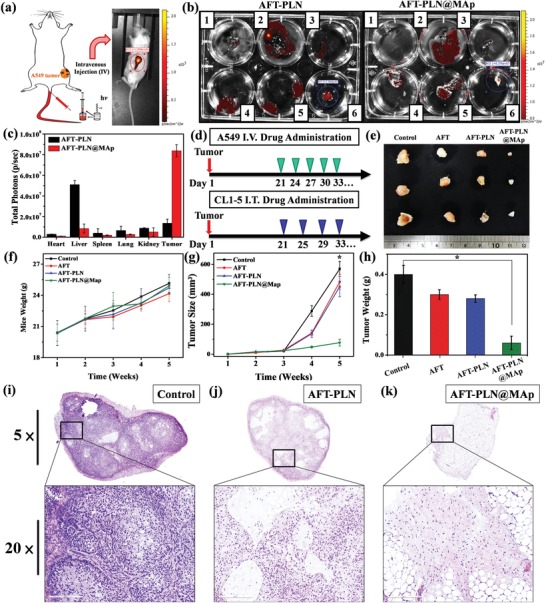
In vivo IV injection to cure SC A549 lung tumors. a) Simulated imaging showing IV drug administration. The material was injected into the mouse through the tail vein after irradiation under the UV light source for 30 min in vitro; the luminescence intensity of PLN was observed by IVIS after 10 min. b) Testing the accumulation of AFT‐PLN and AFT‐PLN@MAp (1: heart, 2: liver, 3: spleen, 4: lung, 5: kidney, 6: tumor). The ROI value shows the luminescence intensity of the c) quantified luminescence data in different organs. This value is calculated using the IVIS ROI test. d) The treatment procedure of drug administration. First, in the IV and IT administration design, A549 and CL1‐5 tumor cells were injected and grew for 3 weeks until the tumor size was nearly 125 mm^3^. In the IV administration experiment, the drug was injected through the tail vein every 3 days. In the IT administration experiment, the drug was administered IT every 4 days. The tumor was removed and observed after the 5th week. e) True tumor size evaluation of the “Control,” “AFT,” “AFT‐PLN,” and “AFT‐PLN@MAp” groups. f) The weight of the mice over 5 weeks. The mouse weight increased from 19 to 25 g. g) Tumor size and h) weight of tumors isolated from mice receiving AFT, AFT‐PLN, and AFT‐PLN@MAp treatment. *n* = 8 (*p < 0.05 compared with the control group). H&E staining of collected tumor tissue by groups i) Control, j) AFT‐PLN, and k) AFT‐PLN@MAp. The full tumor image was obtained with a visual range zoom of 5×, and the 20× images of the tumor revealed the detailed tumor cells (scale bar: 3 µm and 200 nm).

**Figure 7:**
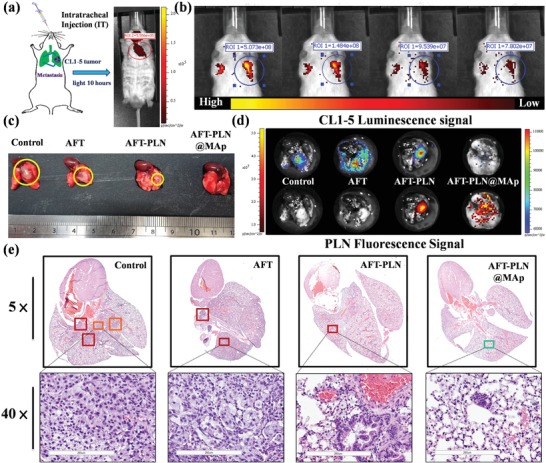
In vivo IT drug delivery to cure an orthotopic CL1‐5 lung tumor. a) Schematic imaging showing IT drug administration. IT experiment: After irradiation with the UV light source for 30 min, the material was injected into the mouse intratracheally, and the luminescence intensity of the PLN was directly observed by IVIS at 10 h. b) IVIS imaging showing the PLN fluorescent signal. The PLN persistent luminescence achieved in vivo at 6 h. Furthermore, the ROI region was chosen to evaluate the intensity of PLN luminescence. c) The true tumor size evaluation by the photo. d) The luminescence of CL1‐5 cells and PLN fluorescent signal. The upper section is the luminescence of cells infected by the lentivirus. The lower section is the fluorescence of the PLN. The tumor and material locations were obtained by luminescence analyses of the IVIS. H&E staining of collected lungs from the e) control, AFT, AFT‐PLN, and AFT‐PLN@MAp groups. The tumor region was obtained with a visual range zoom of 5×, and the 20× images of the tumor revealed the detailed tumor cells (scale bar: 3 µm and 200 nm).

For the animal experiments, we used a material concentration of 10 mg kg^−1^, the drug concentration was 10 µg mL^−1^ and the solution volume was 100 µL for treatment. The luminescence intensity of all materials was evaluated in vitro by IVIS, the came out result was similar to Figure S5g, Supporting Information, and the luminescence intensity was marked by ROIs. **Figures** **6a** and **7a** show the experimental design in the in vivo test. The experiment was divided into two parts: in the first part, A549 lung cancer cells were implanted in the left leg of a mouse via subcutaneous injection on the 3rd week to achieve a tumor the size of 100 mm^3^ and were observed and inspected weekly. The tumors were treated with AFT, AFT‐PLN, or AFT‐PLN@MAp. The treatment began in the 3rd week and was continued once every 3 days by intravenous injection of the drug (Figure 6d). As shown in Figure 6b, AFT‐PLN@MAp clearly accumulates in the tumor area. After dissecting the mice, most of the material was found to accumulate in the liver and kidney, and a small portion accumulated in the lungs. Compared to the AFT‐PLN group, AFT‐PLN@MAp showed significant tumor accumulation (Figure 6c). Moreover, the accumulation of AFT‐PLN and AFT‐PLN@MAp groups was analyzed by inductively coupled plasma mass spectrometry (ICP‐MS) to obtain more precise information on particle position. The mouse contains less of the Ga element; therefore, the difference could be compared using ICP analysis. Figure S6a, Supporting Information, shows the ICP‐MS results of the SC group. It can be observed that the results are consistent with IVIS data, that the content of Ga in the tumor is the highest, followed by the liver and kidney. Mouse weights were measured to determine health, as shown in Figure 6f. Although there was no difference observed in weights, a slight trend was observed in the AFT experimental group; the weight of the mice showed a slight decrease because the AFT IV injection caused mild side effects. However, all the other groups exhibited an increasing trend in weights. These results indicated that AFT‐PLN@MAp had few side effects in mice. After drug administration for 2 weeks, a curable effect was reflected in the tumor size in each AFT group (Figure 6g). The tumor size of the two control groups without any treatment was approximately 590 mm^3^. Compared to the other experimental groups (AFT and AFT‐PLN), tumor size with AFT‐PLN@MAp treatment was considerably inhibited (approximately 80 mm^3^) and presented a better curable effect via active targeting. Mice were sacrificed on the 5th week, and the tumors were excised as shown in Figure 6e. Excellent targeting therapy was exhibited with a reduction in tumor size and weight of almost fourfold (tumor size of 590 mm^3^ in the controls reduced to 75 mm^3^ with AFT‐PLN@MAp and weights from 0.4 to 0.06 g, respectively, Figure 6h). After sacrifice, the tumor tissue was collected and stained with hematoxylin and eosin (H&E). As expected, significant damage was observed in the removed tumors in the AFT‐PLN and AFT‐PLN@MAp groups (Figure 6i–k). The AFT‐PLN@MAp group also had hollow tumors. In addition, the H&E staining results showed that all the organs collected (heart, liver, spleen, lung, and kidney) did not have significant damage or change after AFT, AFT‐PLN, and AFT‐PLN@MAp treatment (Figure S7, Supporting Information). As a biosynthesis enzyme, AK4 is significantly associated with lung cancer metastasis. AK4‐induced lung metastasis occurs due to the downregulation of the transcription factor ATF3. Lung cancer patients with high AK4 expression and low ATF3 expression have poor outcomes. The AK4‐overexpressing CL1‐5 cell line increases lung colony tumorigenesis. In the second part, the CL1‐5 cancer cell lines were infected by luciferase‐carrying lentivirus. This caused CL1‐5 cells to have a luminescence signal that could be detected by IVIS. Orthotopic lung injection is shown in Figure S5h, Supporting Information. With this preliminary result, we designed AFT‐PLN@MAp to accumulate in metastatic CL1‐5 tumor tissues via active transport. To evaluate the luminescence (CL1‐5)/fluorescence (AFT‐PLN@MAp) effect in vivo of AFT‐PLN@MAp, we first collected in vivo NIR persistent luminescence images of mice injected with orthotopic CL1‐5 tumor cells. After irradiation with a UV flashlight source, the NIR persistent luminescence signal was repeatedly observed in the mouse's mouth/lung for at least 6 h after IT treatment with AFT‐PLN@MAp (Figure 7b; the IT treatment process is shown in Figure S8a,b, Supporting Information). Since the particles enter the lung and its tumor cells directly, no significant difference in the NIR persistent luminescence signal was observed in the other parts of the mice, demonstrating that AFT‐PLN@MAp only accumulated in lung tissue. This result was also analyzed by ICP‐MS to quantify and prove the accumulation of the material. It can be seen that the lung has the most Ga element accumulation, while other organs did not show any noticeable signals as shown in Figure S6b, Supporting Information. The lungs were collected after the mice were sacrificed and included the following different groups: AFT, AFT‐PLN, and AFT‐PLN@MAp, as shown in Figure 7c, with the true image (another set of data for immersing lung tissue in formalin is shown in Figure S8c, Supporting Information) and with the IVIS image (Figure 7d). The results showed that the AFT drug had an inhibitory effect on tumors, but the tumor‐suppressing ability at this concentration was limited. The use of AFT‐PLN@MAp had more effective results for tumor suppression. The AFT‐PLN@MAp group revealed whether it was in situ tumor suppression or metastatic tumor suppression, which could accurately inhibit tumor growth and reduce the risk of metastasis. Orthotopic lung experiments involved the injection of CL1‐5 tumors into the left lung, followed by H&E staining for preliminary analysis of normal lung organs and tumor cells. In Figure 7e, the CL1‐5 tumor was treated with AFT, AFT‐PLN, and AFT‐PLN@MAp. The group that did not receive any materials and drugs was the “Control group.” The results showed that the CL1‐5 tumor cells of the control group clearly proliferated in the left lung (red box) and produced metastatic CL1‐5 tumor tissues in the right lung (orange box). AFT effectively inhibited tumor metastasis but did not inhibit the growth of the orthotopic tumors (red box). In contrast, the AFT‐PLN and AFT‐PLN@MAp groups demonstrated increased inhibition of orthotopic tumors, and it was almost impossible to detect the production of CL1‐5 cells in the AFT‐PLN@MAp group (green box). This indicated that AFT‐PLN@MAp, which actively transported drug, could be used as a nano‐targeted drug package that was effective in inhibiting metastatic CL1‐5 tumor cells. In general, the use of these NOD‐SCID mice is due to its lack of immunity, making it easier to observe metastatic phenomena when transplanting NSCLC. If we chose a more suitable murine model of lung cancer in future research that will definitely improve the quality of this work. The interaction between nanoplatforms and the immune system can be comprehended via a suitable model.^[^ ^37^ ^]^

Even though we do our best to optimize the research results to mimic humans, the detection in the larger species such as humans remains a great technical challenge in luminescent assays. To achieve this, materials with optimized luminescent efficiency, high biosafety, and controllable surface chemistry are needed. Not only that, the sensitivity and signal/noise resolution of the instrument also need to be improved. Through the complementation of these two technologies, it is believed that the theranostic technique of nano‐fluorescence can be used in clinical applications.

## 3 Conclusion

Metastasis is the major cause of mortality in lung cancer. However, a developing metastatic symptom is difficult to diagnose. Once the lung cancer patient progresses to the distant metastasis stage, their prognosis is poor. Lung cancer cells grown in situ usually result in invasion and are frequently recurrent after therapy. Therefore, early diagnosis of lung cancer‐induced metastasis is essential. In this study, ZnGa_2_O_4_ (ZGO) persistent luminescence nanomaterials (PLNs), based on the biological characteristics of metastatic lung cancer, were used to promote the clinical application of these nanomaterials for long‐term specific tracking. A silica shell‐assisted synthetic route for mono‐dispersal, near infrared PLNs, was developed and applied in the in vitro cell model and in vivo mouse model. The Cr^3+^‐doped PLNs were able to emit light for longer than several hours. After the optimization of physical and chemical properties, the NIR PLNs absorbed the energy of UV or X‐ray light first and continued to demonstrate luminescence emission. Moreover, we identified the specific aptamer MAGE‐A3 and modified the PLN accordingly to increase its targeting ability. These targeted molecules could track metastatic cells via specific binding. The advancement of nanoscale techniques in lung cancer metastasis is an urgent need. Hence, this research delivers a credible diagnostic strategy and a novel method for therapeutic administration. We showed that the multi‐functional NIR PLN was not only a highly sensitive sensor for lung cancer metastasis but also a therapeutic agent that inhibited metastasis. The targeting drug, AFT, was loaded in mesoporous PLNs to obtain the final product, AFT‐PLN@MAp. In the current research, no PLN material combined with the targeted drugs and specific aptamers to treat lung cancer. This novel nano platform has undergone a series of analyses and evaluations, particularly in vitro and in vivo trials. To evaluate the luminescent effect of NIR PLNs in vitro and in vivo, repeated in vivo NIR persistent luminescence images of a normal mouse after SC and IT injection of NIR PLNs were evaluated. After irradiation with a UV flashlight source for 120 s, the NIR persistent luminescence signal could be repeatedly observed throughout the IV and IT treatment of tumors in mice. Interestingly, no significant difference in the NIR persistent luminescence signal was observed in other parts of mice. The major task was to provide a potential strategy for diagnostic treatment in lung cancer. We hope to contribute safe and multifunctional nanoparticles that can be used to improve the prognosis of lung cancer metastasis. This nanoparticle‐assisted diagnostic and therapeutic strategy is a significant potential treatement method for lung cancer metastasis. The current diagnostic method does not easily distinguish between tumor cells and the metastatic process, with the detection limit being a major issue. Moreover, we expect that our designed nanoplatform or procedure could be applied to other types of materials or cancers and hope that this nanoplatform will lead to nanomaterials that can be used in the exploration of gene and metastatic research.

## 4 Experimental Section

##### Materials

All chemicals were used without purification. The ammonium hydroxide solution (NH_4_OH in H_2_O, 30–33%), methanol (CH_3_OH, 99.99%), and tetraethyl orthosilicate (TEOS, 99.99%) were obtained from Sigma‐Aldrich (Saint Louis, MO, US). Ethanol (C_2_H_5_OH, 99.8%) was purchased from Thermo Fisher Chemical (Waltham, Massachusetts, US). (1‐Hexadecyl) trimethyl ammonium bromide (CTAB, 98%), zinc acetate (Zn(CH_3_COO)_2_, 99.98%), tin(II) chloride (99%), and chromium(III) nitrate nonahydrate (Cr(NO_3_)_3_·9H_2_O, 98.5%) were obtained from Alfa Aesar (Haverhill, MA,USA). Gallium oxide (Ga_2_O_3_, 99.99%) was procured from Gredmann. Nitric acid (HNO_3_, 69–70%) was from J. T. Baker (Phillipsburg, NJ, USA). The Keratinocyte Serum‐Free Growth (K‐SFM) and Roswell Park Memorial Institute (RPMI 1640) media were purchased from Thermo Fisher Scientific (Waltham, Massachusetts, US) for cell seeding. The Beas2B normal lung bronchus cells used K‐SFM medium, and A549 and CL1‐5 lung cancer cells were incubated in RPMI 1640 medium. The penicillin–streptomycin–glutamine (PSG) supplement for culture media was purchased from GIBCO (Waltham, MA, USA).

##### Preparation of MSNs

Hexadecyltrimethylammonium bromide (CTAB), 1 g, was stirred into 465 mL of water with 3.5 mL of 2 m NaOH solution. Subsequently, 5 mL of tetraethylorthosilicate (TEOS) was added slowly (one drop at a time). The solution was then stirred again at 80 °C for 2 h. The MSNs were obtained after washing three times with anhydrous methanol and drying under vacuum oven at 110 °C for 3 h. The white substances were crushed into a powder using a mortar and pestle. The CTAB template was removed by calcination at 550 °C (5 °C min^−1^) for 4 h.

##### Synthesis of PLNs

The 0.25 m Ga(NO_3_)_3_ solution was prepared using 40 mL of nitric acid and 60 mL of water to solve Ga_2_O_3_ (4.5861 g, 0.025 moles) at 95 °C until the Ga_2_O_3_ dissolved completely. The ZGOCS@MSNs (PLNs) were synthesized using an MSN template method with some modifications to limit the size distribution. Typically, 8 mL of Ga(NO_3_)_3_ solution (0.25 m), 183.5 mg of Zn(CH_3_COO)_3_, 0.5 mL of Cr(NO_3_)_3_ solution (0.02 m) and 0.2 mL of SnCl_2_ (0.05 m) were mixed to form a precursor solution. Next, 4 mL of the precursor solution was mixed with 0.5 g of MSN, and the mixture was dried in a vacuum oven at 110 °C for 3 h. The dried sample was crushed using a mortar and pestle and was pre‐sintered at 600 °C for 2 h with a heating rate of 5 °C min^−1^. The heated mixture was taken out and ground into a fine powder. The pre‐sintered material was annealed at 1000 °C for 4 h with a slow heating rate of 2 °C min^−1^. Finally, the PLNs were obtained after cooling down to room temperature.

##### Drug Loading (AFT‐PLN)

PLNs (1 mg) was added to 1 mL of AFT DMSO/ethanol solution (0.5 mg mL^−1^), and the mixture was stirred overnight. The dispersion was centrifuged and washed with DMSO three times. The supernatant was collected after the loading process, and the absorption was analyzed and compared to a pre‐determined standard curve of AFT. The amount of AFT loaded and loading efficiency was determined. The loading efficiency in wt% was computed as follows:

(1) $$\text{Drug}\;\text{loading}\left(\%\right)=\frac{\text{Mass}\;\text{of}\;\text{drugs}\;\text{incorporated}\;\text{into}\;\text{particles}}{\text{Mass}\;\text{of}\;\text{particles}}\times 100$$

The loading amount of AFT in AFT‐PLN was confirmed by thermogravimetric analysis (TGA) and UV–vis spectra. For TGA analysis, we synthesized 5 mg of materials and heated those groups at 500 °C to ensure all the organic composed drug and oligonucleotides will degrade to CO_2_ under an oxygen environment. The weight loss will be calculated, and the different heating regions will be defined based on the drug loading and aptamer modification. Except for TGA analysis, the UV–vis spectrum can also be used to evaluate the drug loading and release. AFT has a specific absorbance at approximately 200 nm and can be detected by UV–vis. The release curve can be illustrated according to the record of the UV–vis spectrum.

##### Surface Modification of AFT‐PLNs (AFT‐PLN@MAp)

By the Michael addition reaction, the surface of AFT‐loaded PLNs (AFT‐PLNs) was conjugated to the functionally targeted ligand MAp.^[^ ^38^, ^39^ ^]^ Thus, the PEGylated aptamer (H_2_N‐PEG‐MAp) was prepared first. Briefly, 10 OD MAp‐SH was resolved by 500 µL of Tris‐HCl (10 mm, pH ≌ 7.4). To prevent oxidation of the thiol group, 500 µL of MAp‐SH solution was added to 1 mg of NH_2_‐PEG‐MAL accompanied by 20 µg of TCEP, followed by mixing the solution for 3 h. The resulting H_2_N‐PEG‐MAp was successfully synthesized. Next, H_2_N‐PEG‐APt was added to AFT‐PLNs, and Tris buffer (10 mm, pH 8.5) was utilized to resuspend the mixture. After stirring in the dark for 3 h, the resultant compounds considered as AFT‐PLN@MAps were subjected to centrifugation, washing three times with deionized water as well as drying in a vacuum oven. Thus, AFT‐PLN@MAps were functionalized with aptamer, which was the targeted ligand.

##### DNA PAGE Analysis

Polyacrylamide gels are used to separate shorter nucleic acids, generally in the range of 1–1000 base pairs, based on the concentration used. In this study, 10% gels were chosen to separate the aptamer. Regarding the positive and negative controls, the MAp was compared with the other the 26‐mer active site MAP (positive control) and 52‐mer PB (negative control). Moreover, the AFT‐PLN@MAp was added to evaluate the aptamer conjugation.

##### Western Blot Analysis

Equal quantities of protein (20 µg) were separated using SDS‐PAGE and were transferred to the nitrocellulose membranes (Amersham Bioscience, Buckinghamshire, UK) using a Bio‐Rad wet transfer system. After blocking with 5% non‐fat dried milk in PBS/Tween20 buffer for 1 h at room temperature, the membranes were incubated with the MAGE‐A3 antibody for 1 h at room temperature to detect the expression amount of different lung cell proteins, including Beas2B (normal lung cells), H1355 (adenocarcinoma), A549 (carcinoma), CL1‐0 (adenocarcinoma), and CL1‐5 (metastatic adenocarcinoma). Thereafter, the membranes were incubated with horseradish peroxidase‐conjugated secondary antibody for 1 h at room temperature. Proteins were visualized using enhanced chemiluminescence. After the evaluation of western blotting, we chose Beas2B, A549, and CL1‐5 as the model experimental cells.

##### Cellular Uptake and Localization Analysis

Approximately 20 000 cell lines of Beas2B, A549, and CL1‐5 per mL were planted on a well plate slide for 12 h, while a 250 µg mL^−1^ nanoparticle composite was added to the culture at 37 °C with 5% carbon dioxide for 12 h. The cells were washed with 10 mm phosphate‐buffered saline (PBS; pH 7.4) and fixed in 4% paraformaldehyde fixative (paraformaldehyde) to maintain their intrinsic form. Subsequently, the nuclear stain DAPI was added for nuclear staining. After 5 min of incubation, the dye was removed and observed by laser scanning confocal microscopy (LSCM). The nucleus can be excited by a 408 nm UV laser, while the emission image can be detected at 450–500 nm. The nanocomposite can also be excited by a UV laser, while its emission is detected at 700–750 nm. The LSCM imaging involved DiI membrane staining. To recognize the binding effect between different nanocomposites, we used DiI dye to localize the place of nanoparticles.

##### In Vitro Cell Viability And Cytotoxicity Analysis

This study selected the Beas2B normal lung, A549, and CL1‐5 lung cancer cell lines as the observation objects of material biocompatibility. The K‐SFM medium added pituitary fluid and epidermal growth factor to maintain the growth of Beas2B cells. By contrast, the RPMI medium with 1% PSG was mixed with 10% fetal bovine serum as the culture solution for A549 and CL1‐5 cells. These cell lines were cultured at 37 °C and 5% carbon dioxide. Approximately 2000 cell lines were cultured in 96‐well plates for 12 h, while nanocomposites of 3, 9, 27, 81, and 250 µg mL^−1^ were added to the individual wells for 24 h. The cell dye Alamar Blue was likewise added. Cell staining was performed, while the fluorescence intensity of Alamar Blue was detected by a fluorophore to quantify the cell viability and cytotoxicity.

##### Flow Cytometric Analysis

Beas2B, A549, and CL1‐5 lung cells (2 × 10^5^ cells) were exposed to the nanocomposite (100 µg mL^−1^) for 48 h, detached by trypsin‐EDTA, and washed with PBS. The active targeting ability was analyzed by flow cytometry using FAC Scan. The results were investigated using CellQuest software. Depending on the emission of PLNs, the filler peridinin chlorophyll protein complex (PerCP‐A, it is excited by a 488 nm argon laser, and with a relatively large Stokes shift, emits at a wavelength of 675–750 nm) was chosen to estimate the luminescence of PLN.

##### In Vivo Therapeutic Effect

Animal experiments were approved by the Institutional Animal Care and Utilization Committees of Academia Sinica (IACUC NO. 16‐05‐957). Male age‐matched NOD‐SCID mice (supplied by LASCO, Taiwan) between 6 weeks old were used to assess tumor growth in lung metastasis models. Two different drug treatments were set up. In the first experiment, to evaluate the targeting therapeutic ability of AFT‐PLN@MAp, the A549 lung cancer cell line (5 × 10^6^ cells/100 µL PBS) was injected subcutaneously into the right thigh of the mouse. The AFT (drug), AFT‐PLN, and AFT‐PLN@MAp nanocomposites (100 mg) were injected by IV injection when the tumors had grown to 125 mm^3^ (approximately 3 weeks) and the tumor in mice was observed by IVIS spectrum. Thereafter, all tumors were acquired after another 2 weeks (the total duration of the animal experiment was 5 weeks). In the treatment design, the mice were first continuously treated with the drug material by IV injection every 3 days. The second experiment was used to optimize the therapeutic effect of AFT‐PLN@MAp, and the CL1‐5 lung cancer cell line (1 × 10^6^ cells/10 µL PBS) was mixed with the same volume of Matrigel (Matrigel:PBS at 1:1 ratio) injected orthotopically into the left lung of the mouse. About 80% of the mice successfully grew tumor cells and produced metastasis phenomena (*n* = 10 mice were used, and *n* = 8 were selected for statistics). Moreover, lung metastases were traced with bioluminescent imaging every 2 days by IVIS after treatment with following groups: the AFT (drug), AFT‐PLN, and AFT‐PLN@MAp nanocomposites (100 mg) were injected by IT injection after the tumors had grown approximately 3 weeks, and the tumor in mice was observed using the IVIS spectrum. A different time was chosen to observe the luminescence of PLNs for 1 to 6 h. Thereafter, all lung tissues were acquired after another 2 weeks (the total duration of the animal experiment was 5 weeks), and the IVIS system was used to estimate the therapeutic results of AFT‐PLN@MAp.

##### Tissue Staining and ICP‐MS Analysis

The tumor and organ sections were formalin‐fixed and paraffin‐embedded. The cross‐sections of the tumor were stained using hematoxylin and eosin (H&E staining). The lung and other organs tissue sections were baked in an oven for 10 min and dewaxed with xylene and ethanol. After washing in PBS for 3 min, the sections were soaked in hematoxylin for 1 min. After adding ammonia for 20 s, tissue sections were washed in the PBS for 6 min to strengthen the staining; then soaked in eosin for 2 min. Finally, the tissue sections were sealed after dehydration through xylene and ethanol. All the stained tumor sections were observed using a Leica Aperio AT2 scanner. For the ICP‐MS (X series II, Thermo) analysis, the Ga element was chosen to confirm the accumulation and composition of the host material, ZnGa_2_O_4_ (PLN). 20 mg tissue of each organ and tumor were dissolved in nitric acid at 60 °C overnight. After the tissue dissolved, the buffer was used to dilute the sample solution with a concentration of 1:10 000. The diluted sample solution was passed through the Millex‐GN filter (0.2 µm, nylon 13 mm) to remove impurities and obtain the analytical sample for ICP‐MS.

## Conflict of Interest

The authors declare no conflict of interest.

## Supporting information

Supporting Information
