# Supplementary material for: Next‐Generation Cancer‐Specific Hybrid Theranostic Nanomaterials: MAGE‐A3 NIR Persistent Luminescence Nanoparticles Conjugated to Afatinib for In Situ Suppression of Lung Adenocarcinoma Growth and Metastasis
Source: Adv Sci (Weinh). 2020 Mar 14;7(9):1903741. doi: 10.1002/advs.201903741 (PMC7201263; doi:10.1002/advs.201903741)
Supplement: Supplementary file 1 — Supporting Information [file ADVS-7-1903741-s001.pdf]

Copyright WILEY-VCH Verlag GmbH & Co. KGaA, 69469 Weinheim, Germany, 2020.

Supporting Information

for *Adv. Sci.,* DOI: 10.1002/advs.201903741

Next-Generation Cancer-Specific Hybrid Theranostic Nanomaterials: MAGE-A3 NIR Persistent Luminescence Nanoparticles Conjugated to Afatinib for In Situ Suppression of Lung Adenocarcinoma Growth and Metastasis

Ming-Hsien Chan, Wen-Tse Huang, Jing Wang, Ru-Shi Liu,* and Michael Hsiao**

Copyright WILEY-VCH Verlag GmbH & Co. KGaA, 69469 Weinheim, Germany, 2020.

Supporting Information

Next Generation Cancer-Specific Hybrid Theranostic Nanomaterials: MAGE-A3 NIR Persistent Luminescence Nanoparticles Conjugated to Afatinib for in-situ Suppression of Lung Adenocarcinoma Growth and Metastasis

Ming-Hsien Chan, Wen-Tse Huang, Jing Wang, Ru-Shi Liu*, and Michael Hsiao**

Supplementary Figure 1

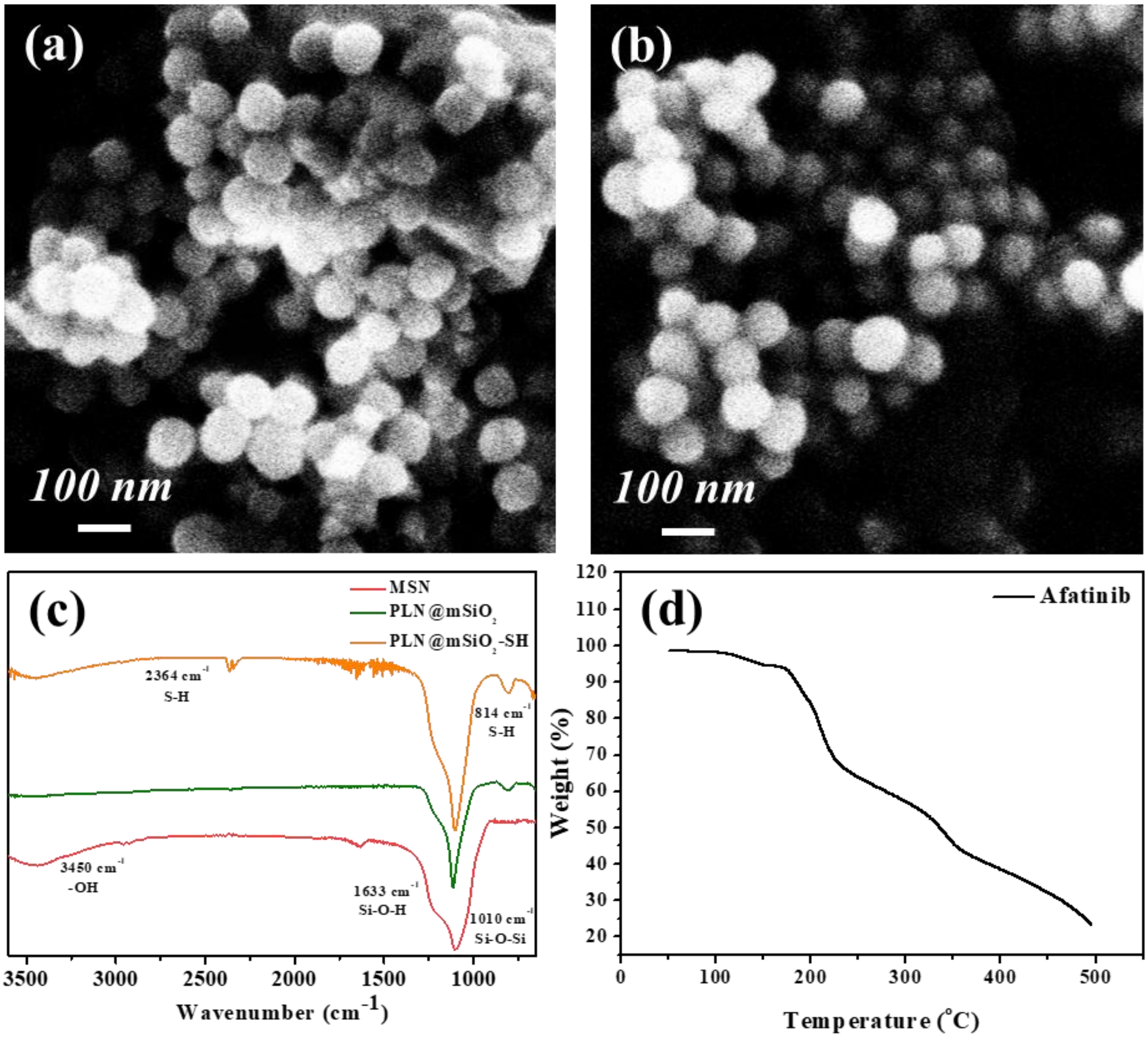

Figure S1. The characterization of MSN and AFT-PLN@Map. The morphology of (a) MSN and (b) AFT-PLN@MAp can be observed using a scanning electron microscope (SEM). (c) FTIR spectrum detection the -SH thiol functional group has been successfully conjugated with PLN. (d) The TGA curve of AFT shows the trend is similar to the group of AFT-PLN@MAp.

Supplementary Figure 2

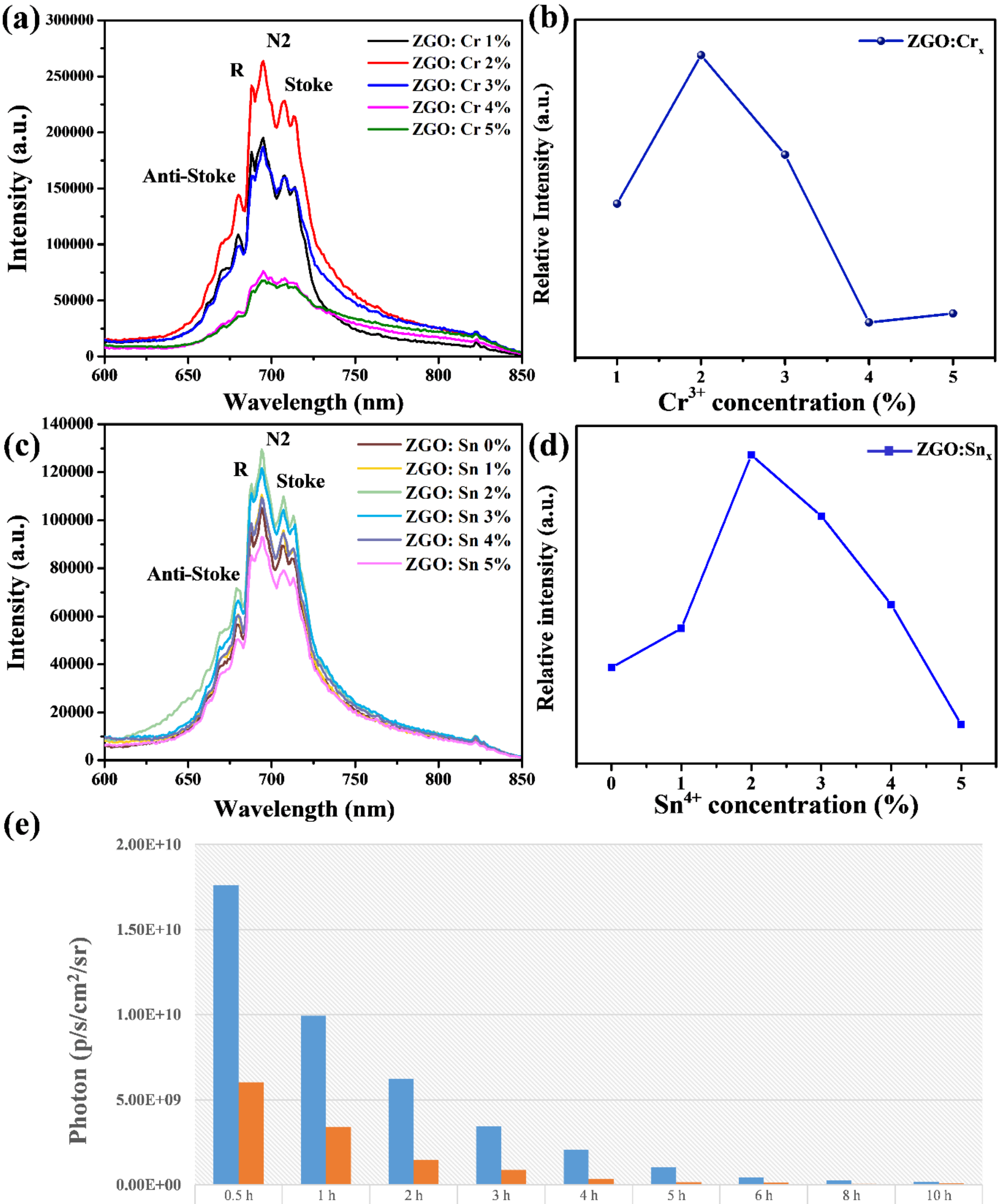

	0.5 h	1 h	2 h	3 h	4 h	5 h	6 h	8 h	10 h
ZnGa2O4:Cr3+,Sn4+	1.76E+10	9.94E+09	6.24E+09	3.45E+09	2.08E+09	1.03E+09	4.31E+08	2.63E+08	1.80E+08
ZnGa2O4:Cr3+	6.03E+09	3.42E+09	1.47E+09	8.88E+08	3.59E+08	1.64E+08	1.27E+08	6.63E+07	8.64E+07

Figure S2. The optical properties optimization test for PLN. Different concentration and the photoluminescence intensity of (a) Cr^{3+} and (c) Sn^{4+} ions in PLNs. The photoluminescence intensity trend diagram for controlling the concentration of the (b) Cr^{3+} and (d) Sn^{4+}precursor solution in the PLNs. (e) The photons quantification value of ZGOCS and ZGOC are imaged individually in the IVIS Spectrum based on Figure 3e. Those experimental results are depended on the same ROI measurements in Figure 3e.

Supplementary Figure 3

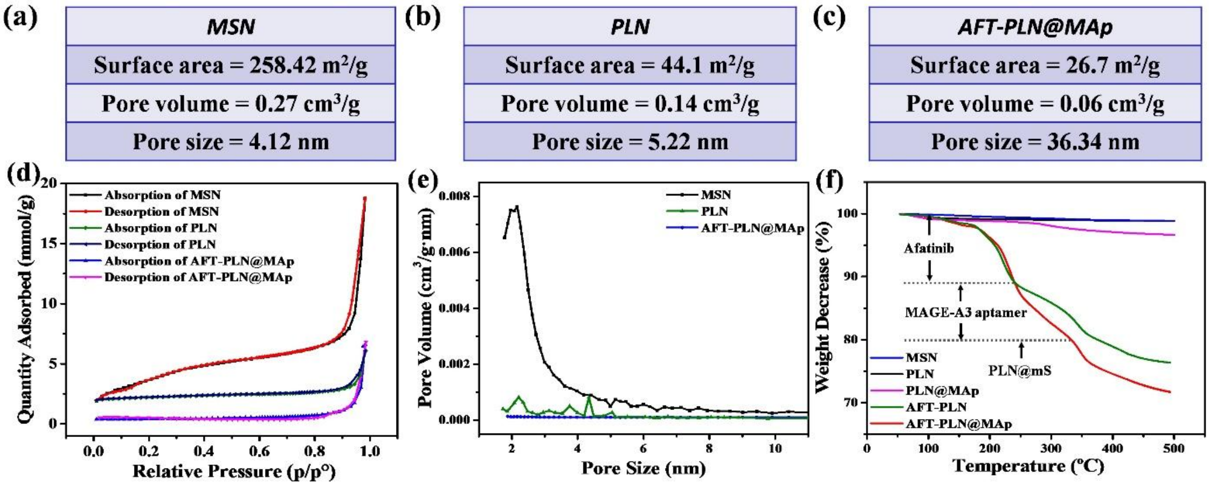

Figure S3. Mesoporous and drug-loading evaluation. The BET quantified data of (a) MSN and (b) PLN and (c) AFT-PLN@MAp and the BET (d) isothermal curve and (e) pore evaluation of MSN and AFT-PLN@MAp. The BET isothermal curve shows that the pore structure of the MSN is a hole in Type IV with a pore size of approximately 2.2 nm. Compared with AFT-PLN@MAp, the hysteresis loop of the mesoporous structure disappears and the aperture is almost zero. (f) TGA analysis of MSN, PLN, PLN@MAp, AFT-PLN, and AFT-PLN@MAp. The sections of the curve show the weight loss results after AFT and aptamer combustion at high temperatures.

Supplementary Figure 4

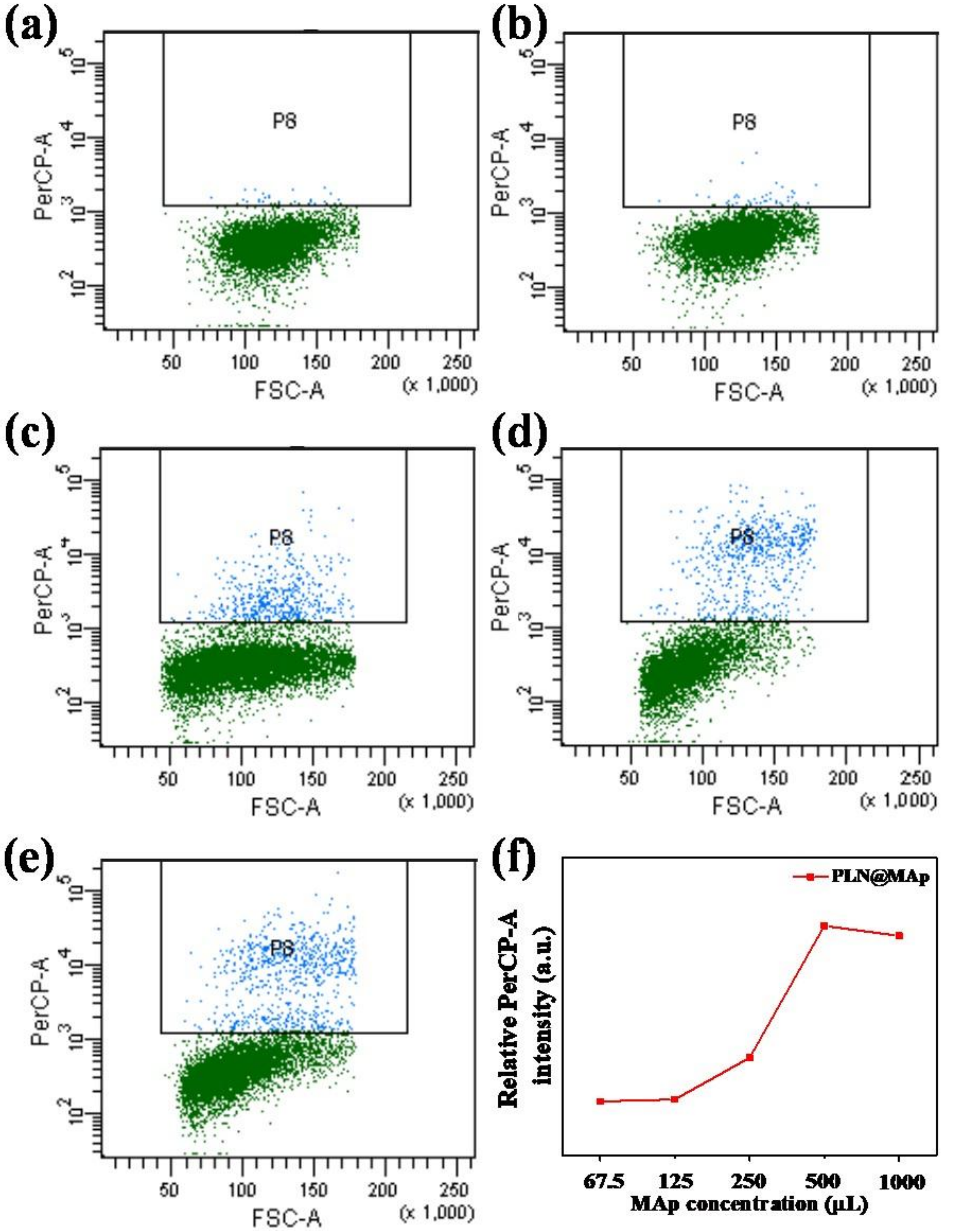

Figure S4. Flow cytometry of different concentrations of Map. PLN@MAp-modified with MAp from (a) 67.5 μL, (b) 125 μL, (c) 250 μL, (d) 500 μL to (e) 1 mL. Those samples are treated with A549 cell lines. The left columns are the cell point distribution map, and the right columns are the fluorescence of the cells. (e) The relative curve quantified the P8 fluorescent region.

Supplementary Figure 5

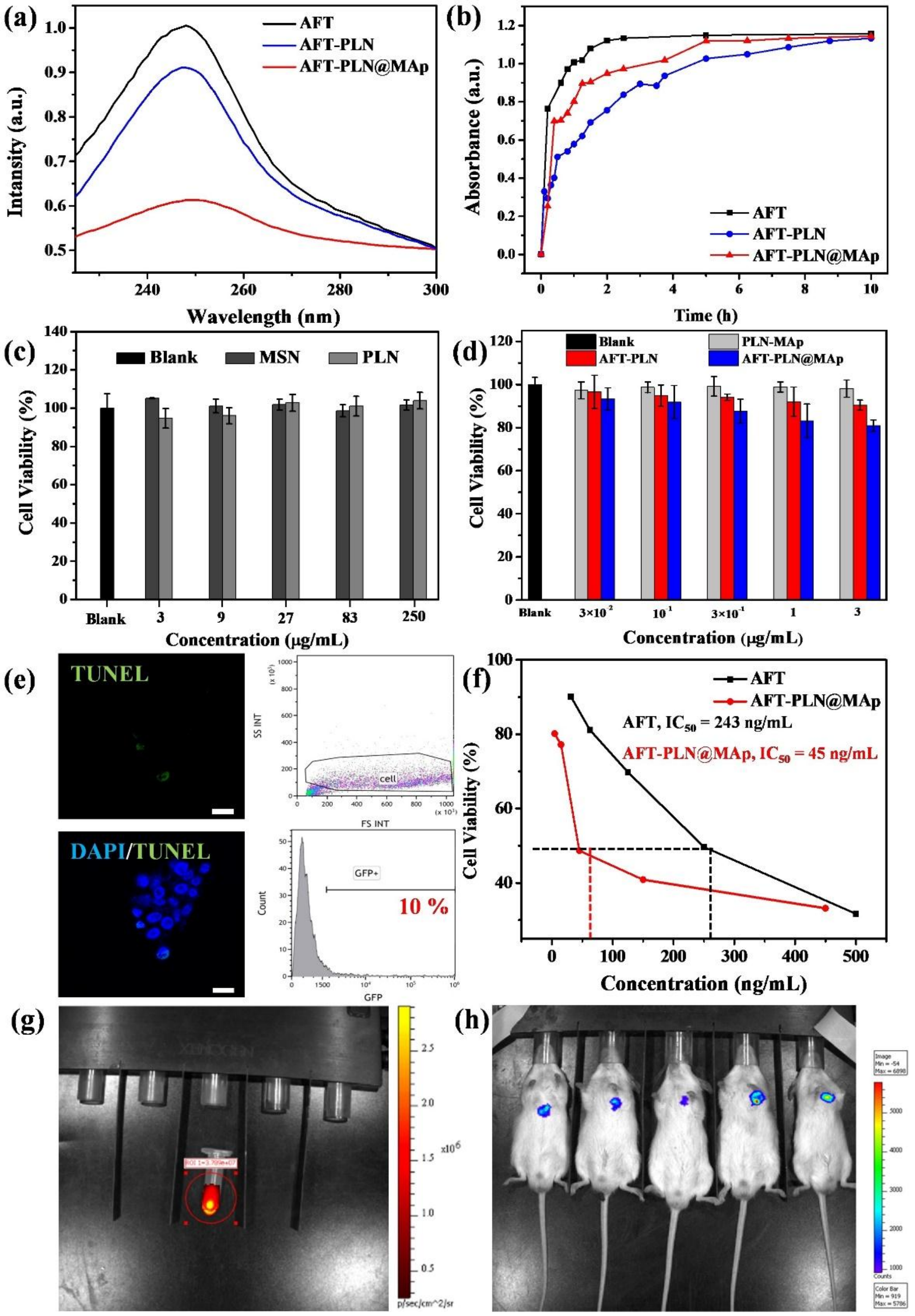

Figure S5. Cell and biological imaging analysis. (a) UV spectra of AFT, AFT-PLN, and AFT-PLN@MAp for 1 hour drug release. (b) The drug releasing-curve of AFT, AFT-PLN, and AFT-PLN@MAp. The cytocompatibility test. The (c) A549 and (d) Beas2B cells can be found all have survival values higher than 80%. (e) TUNEL assay and flow analysis of PLN (DAPI: 440 nm emission and TUNEL: 520 nm emission). (f) Drug toxicity analysis. The data of **Figure 4a** and **4b** in this study are plotted to obtain the IC50 values. (g) Fluorescent images obtained by AFT-PLN@MAp under IVIS examination. (h) Mouse orthotropic lung tumor implantation. The CL1-5 cell line was infected with luminescence lentivirus and the cells have the luminescent signal to obtain the true location of the tumor.

Supplementary Figure 6

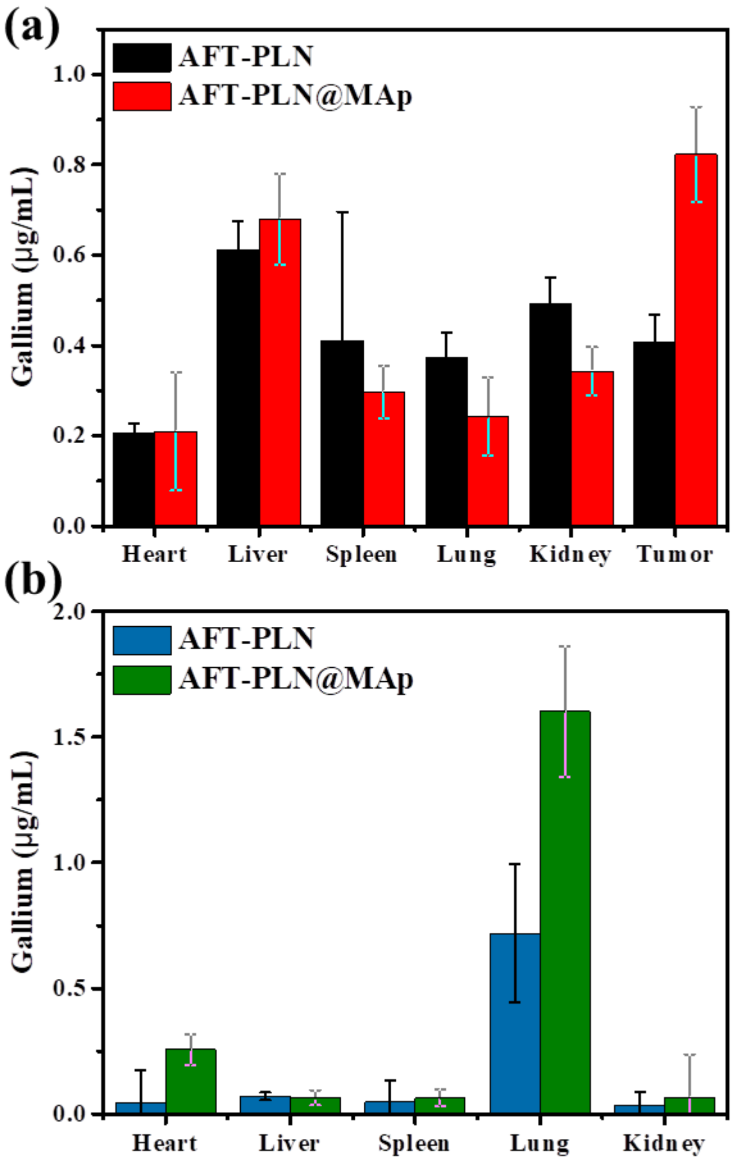

Figure S6. Statistical analysis of ICP-MS data of the mice organs. Comparison of ICP-MS indicated Ga^{3+} ions with (a) I.V. injection AFT-PLN and AFT-PLN@MAp to calculated ion concentrations of S.C. tumors in different organs. (b) I.T. injection AFT-PLN and AFT-PLN@MAp to calculated ion concentrations of orthotopic lung tumors treatment in different organs.

Supplementary Figure 7

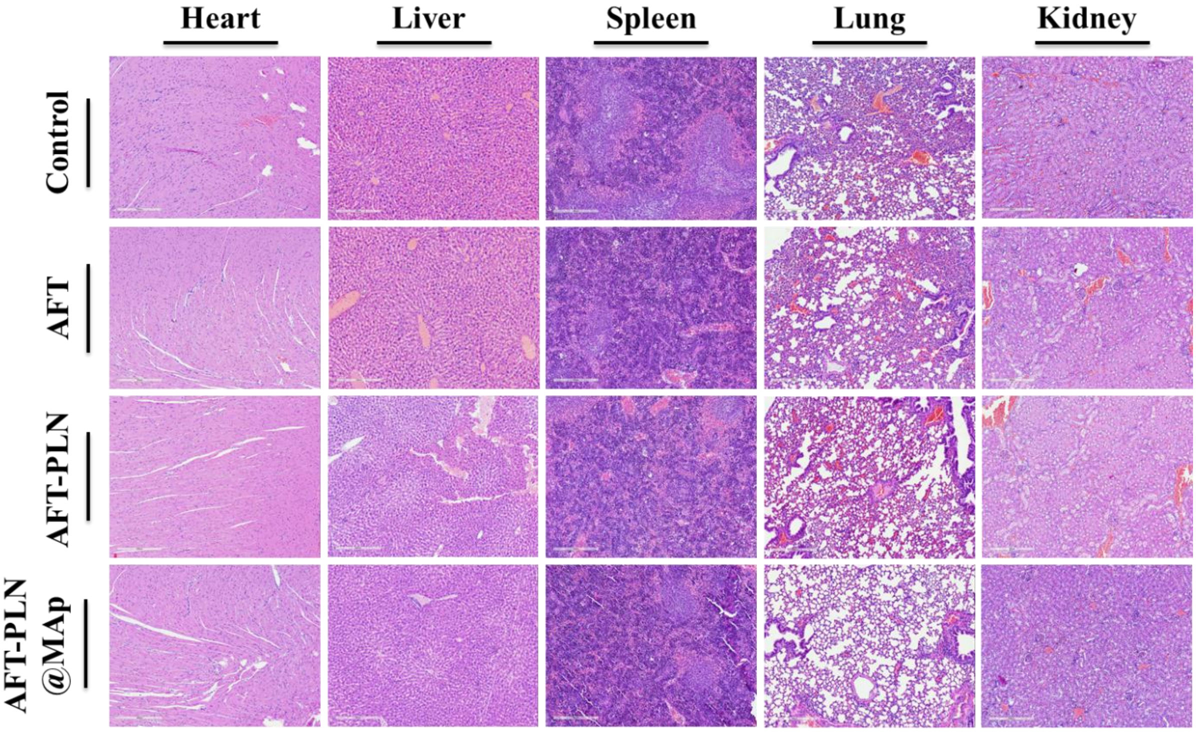

Figure S7. Histopathologic analyses of H&E-stained tissue. Heart, liver, spleen, lung, and kidney organs analysis for tumor-bearing mice after the indicated treatment with AFT, AFT-PLN, and AFT-PLN@MAp, scale bar: 300 μm.

Supplementary Figure 8

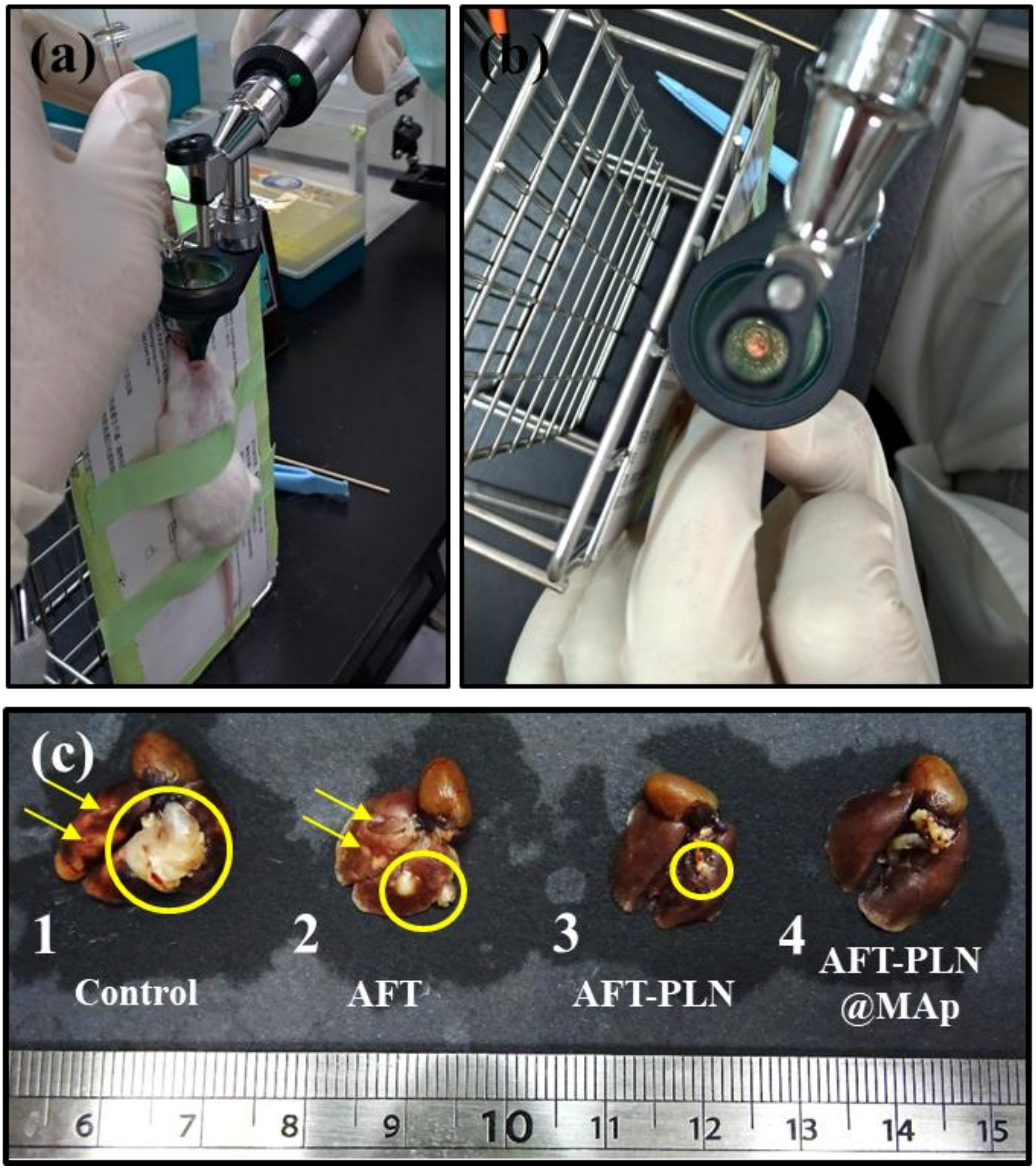

Figure S8. ***In vivo*** **lung organ evaluation.** (a) The I.T. treatment process and (b) The actual photo of mouse tracheal opening. (c) The true tumor size evaluation with different treatment groups. All of the heart and lung tissues were soaked in formaldehyde and revealed obvious contrast between normal lung tissues and tumors.
